# Oligodendrogliomas: findings after classifying the same cohort using pre- and post-World Health Organization (WHO) 2021 criteria

**DOI:** 10.1093/braincomms/fcaf265

**Published:** 2025-08-01

**Authors:** Maria Angeles Vaz-Salgado, Juan M Sepulveda, Julie Earl, Jacqueline Gutierrez, Yolanda Ruano, Hector Pian, Diana Cantero, Aurelio Hernández-Lain

**Affiliations:** Medical Oncology Department, Hospital Universitario Ramón y Cajal, Madrid 28034, Spain; Biomarkers and Personalized Approach to Cancer Group (BioPAC), Area 3, Ramón y Cajal Health Research Institute (IRYCIS), 28034 Madrid, Spain; The Biomedical Research Network in Cancer (CIBERONC), Avda. Monforte de Lemos, 5, 28029 Madrid, Spain; Medical Oncology Department, 12 de Octubre University Hospital, Av Cordoba s/n, 28041 Madrid, Spain; Biomarkers and Personalized Approach to Cancer Group (BioPAC), Area 3, Ramón y Cajal Health Research Institute (IRYCIS), 28034 Madrid, Spain; The Biomedical Research Network in Cancer (CIBERONC), Avda. Monforte de Lemos, 5, 28029 Madrid, Spain; Neuropathology Unit, Pathology Department, imas12 Research Institute, Hospital Universitario 12 de Octubre, Universidad Complutense de Madrid, Av Cordoba s/n, 28041 Madrid, Spain; Neuropathology Unit, Pathology Department, imas12 Research Institute, Hospital Universitario 12 de Octubre, Universidad Complutense de Madrid, Av Cordoba s/n, 28041 Madrid, Spain; Department of Pathology, Ramón y Cajal University Hospital, IRYCIS, Madrid 28034, Spain; Neuropathology Unit, Pathology Department, imas12 Research Institute, Hospital Universitario 12 de Octubre, Universidad Complutense de Madrid, Av Cordoba s/n, 28041 Madrid, Spain; Neuropathology Unit, Pathology Department, imas12 Research Institute, Hospital Universitario 12 de Octubre, Universidad Complutense de Madrid, Av Cordoba s/n, 28041 Madrid, Spain

**Keywords:** oligodendroglioma, CNS WHO 2021 classification, brain tumours, 1p/19q deletion, IDH mutations

## Abstract

The 2021 World Health Organization (WHO) classification includes the presence of isocitrate dehydrogenase (IDH) mutation and 1p/19q codeletion for oligodendrogliomas. The objective of this study was to evaluate the impact of the introduction of this classification in a cohort of oligodendrogliomas. A total of 182 cases with an initial diagnosis of oligodendroglioma by histological criteria were identified, including Grades 2 and 3 and oligoastrocytoma (initial cohort). Subsequently, IDH mutation and 1p/19q codeletion were determined and were present in a total of 91 cases (reclassified cohort). The clinical evolution of both cohorts was analyzed. The mean age was 45 years (14–75), 65% were Grade 2 and 22% were oligoastrocytomas. Complete resection was performed in 47% and biopsy in 7%. After surgery, 50% received radiotherapy, 30% chemotherapy and 36% did not receive adjuvant therapy. In the reclassified cohort, there were no statistically significant differences between Grade 2 and Grade 3 oligodendrogliomas, the median overall survival (OS) in Grade 2 was 13.3 years [95% confidence interval (CI) 8.2–18.4] and 12 years in Grade 3 (95% CI 5.6–18.3). However, in the initial cohort, significant differences were found according to tumour grade. Even in cases without adjuvant treatment, the median OS was 12 years. Compared with this data, the median OS for the cohort that did not meet IDH mutation and 1p/19q codeletion criteria was 7.52 years (95% CI 4.67–10.38). Molecular classification allows a more accurate selection of oligodendrogliomas and implies cases with a better prognosis, regardless of the grade and treatment received. These data should be taken into account in clinical practice and clinical trials.

## Teaser

Vaz-Salgado *et al.* reported the evolution of the same oligodendroglioma cohort before and after the World Health Organization (WHO) 2021 classification. Isocitrate dehydrogenase (IDH) mutation and 1p/19q codeletion lead to a more accurate diagnosis. These cases have a better outcome and tumour grade loses its prognostic impact.

## Graphical Abstract

**Graphical Abstract:**
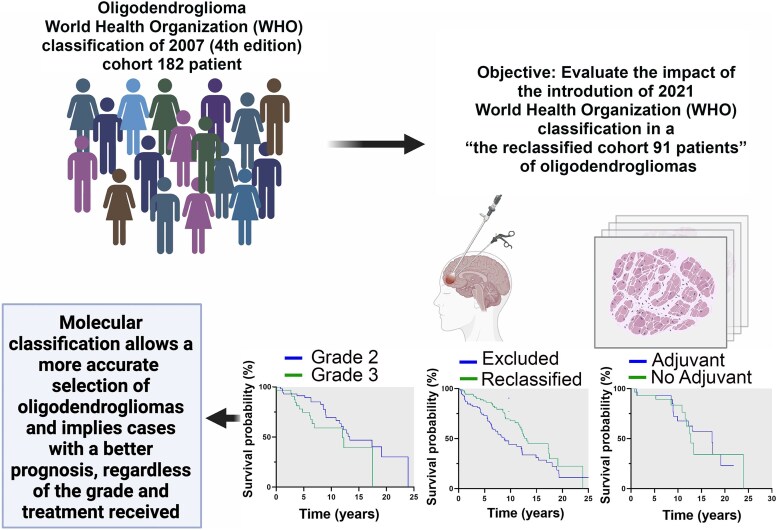

## Introduction

Oligodendrogliomas are rare tumours accounting for 2–3% of primary CNS tumours.^1^ The current 2021 WHO classification of CNS tumours (fifth edition) is built on the previous edition, published in 2016 (revised fourth edition), which for the first time incorporated molecular parameters in addition to the histopathologic appearance to obtain the diagnosis of brain tumours.^2,3^ To establish the diagnosis of oligodendroglioma, the 2021 WHO classification (and the revised fourth edition of 2016) requires the presence of both isocitrate dehydrogenase (IDH) mutations and 1p/19q codeletion.

Treatment recommendations have changed over the years based on the results of different studies in this tumour type, which in addition, have included patients based only on histopathological criteria. The impact of this new classification in clinical practice is not completely described, as this molecular classification provides a better characterization of oligodendroglial tumours and excludes other histological types with an oligodendroglial morphological appearance. The clinical course, risk of recurrence, and treatment considerations of oligodendrogliomas diagnosed based on the CNS 2021-WHO classification have not been fully described. Furthermore, new clinical trials applying these new diagnostic criteria will take time to develop in a tumour with such a low incidence and long survival, so retrospective analysis of case series can provide information for clinical decision-making.

This study aimed to determine the clinical evolution in the same cohort of oligodendrogliomas both before and after applying the new classification criteria, to verify what impact this new classification has on prognosis and therapeutic decisions. In addition, the treatment recommendations in the situation of progression to initial treatment, where there is no standard of treatment were also analyzed.

## Materials and methods

Retrospective cohort study was performed in which patients with a diagnosis of oligodendroglioma were identified from registries in the pathology departments of two tertiary hospitals, corresponding to a period of 37 years, between 1977 and 2014. The initial diagnosis of these cases was made according to diagnostic criteria from the previous WHO classification of 2007 (fourth edition), based solely on histopathological aspects. The search criteria included the diagnoses of oligodendroglioma and oligoastrocytoma (Grade 2 and Grade 3).

This series of cases was named ‘the initial cohort’. Subsequently, a new classification of the same cases was made following the current WHO criteria established in 2021 (fifth edition), which includes IDH mutation and codeletion of 1p/19q.

IDH mutations were identified by High-Resolution Melting analysis and complementary analysis by immunohistochemistry (IHC). Codeletion of 1p/19 q was performed by FISH. A total of 91 cases met the criteria for IDH mutation and 1p/19q codeletion. This cohort was named ‘the reclassified cohort’. On the other hand, the series of patients from the initial cohort who did not meet the criteria for IDH mutation and 1p/19q codeletion were referred to as ‘the excluded cohort’.

Information was obtained on the clinical course of all identified cases, with parameters including date and treatment at diagnosis, time to first recurrence, treatments received after first recurrence, and overall survival (OS).

In this retrospective clinical study, missing values were handled with a pairwise analysis: when a variable was missing for a given case, that particular case was simply excluded from the analysis for that specific variable. Loss to follow-up was minimized by contacting all cases by phone. All cases were followed up until March 2020.

The study has been approved by the local Ethics Committee and consent was obtained according to the Declaration of Helsinki.

### Statistical analysis

Statistical analyses were performed as follows: association study of dichotomous qualitative variables using the Chi-Square test, the survival study was performed using Kaplan–Meier curves. A univariate OS analysis was performed using the Kaplan–Meier method, with log-rank comparisons between the different groups (Supplementary Fig. 1). A multivariate Cox regression model was then performed using the Forward Stepwise (Conditional LR) method. Statistical significance was set at *P* < 0.05.

Raw data are available by contacting the corresponding author.

## Results

A total of 182 patients met the diagnosis criteria based on histopathologic appearance (initial cohort). Of these, 91 met the diagnosis criteria established by WHO 2021, which included IDH mut and 1p/19q codeletion (reclassified cohort). In 19 cases, there was no tissue for the IHC or molecular study, in 24 cases (13.8%), it did not meet the criteria of IDH mutation, and in 58 cases (33.5%) did not meet the criteria of 1p/19q loss.

### Initial cohort

The characteristics of the initial cohort were as follows: 119 cases were Grade 2 (65.4%) and 63 cases (34.6%) were Grade 3; 142 cases were considered oligodendrogliomas (78%) and 40 cases as oligoastrocytomas (22%). There were 70 females (38.5%) and 112 males (61.5%) and the mean age at diagnosis was 44.8 years (14–75). By location, 109 cases were in the frontal lobe (59.9%), 27 cases in the temporal lobe (14.8%), 14 cases in the parietal lobe, and 32 cases in other locations (17.5%). Regarding treatment, only a biopsy was performed in 13 cases (7.1%), partial resection in 36 cases (19.8%), subtotal resection in in 40 cases (22%), and complete resection in 85 cases (46.7%). In eight cases, it was not possible to know the extent of the surgery based on the data collected in the clinical report. After surgery, half of the patients received RT, 58 patients received CT (31.9%) and 33 patients received RT and CT (18%). In 36% of cases, no further treatment was administered.

**Figure 1:**
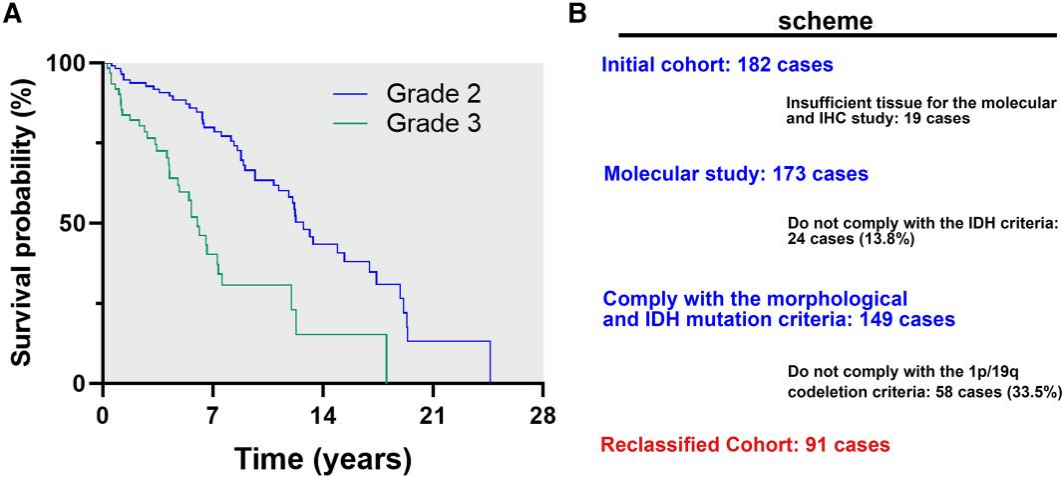
**(A) Median overall survival of the initial cohort according to tumor grade.** Median overall survival for Grade 2 oligodendrogliomas (119 cases) in the initial cohort was significantly greater than for Grade 3 oligodendrogliomas (63 cases); 13.06 years (10.49–15.63) and 6 years (4.9–7.095), respectively (*P* < 0.01). (**B**) Scheme of the reclassification process of the initial cohort, for the definition of the reclassified cohort. Scheme of the reclassification process from initial cohort to reclassified cohort. A retrospective cohort of 182 patients with a diagnosis of oligodendroglioma were identified from two tertiary hospitals from 1977 to 2014, was designated the ‘initial cohort’. This cohort was reclassified following the current fifth edition WHO criteria established in 2021, which includes IDH mutation and codeletion of 1p/19q and the 91 cases in the reclassified cohort were subsequently analyzed.

The median OS for the entire initial cohort was 11.97 years (95% CI 9.76–14.26), and the median progression-free survival (PFS) was 5.69 years (95% CI 4.81–6.57). The median OS for Grade 2 oligodendrogliomas was 13.06 years (10.49–15.63) and 6 years (4.9–7.095) for Grade 3 oligodendrogliomas, *P* < 0.01 (Fig. 1A).

The median OS for oligodendrogliomas was 11.79 years (95% CI 8.74–14.84) and 12.2 years (95% CI 7.86–16.54) for oligoastrocytomas, which was not statistically significant (*P* = 0.560). This initial cohort was reclassified, according to the outline shown in Fig. 1B. The 91 cases in the reclassified cohort were subsequently analyzed.

### The clinical characteristics of the cohort reclassified by WHO criteria 2021

Of the 91 cases analyzed, 59 cases were Grade 2 (64.8%) and 32 cases were Grade 3; the original diagnosis, based on the previous 2007 WHO classification, in which molecular criteria were not included, was oligodendrogliomas in 79 cases (87%) and 12 cases were oligoastrocytoma (13%). There were 31 females (34.1%) and 60 males (65.9%), with a mean age of 44.67 years (22.6–75.2 years). The location of the tumour was the frontal lobe in 69% of the cases, temporal in 12%, parietal in 6.6%, occipital in one case, and other location in 11%.

### Treatments of the reclassified cohort

**Figure 2:**
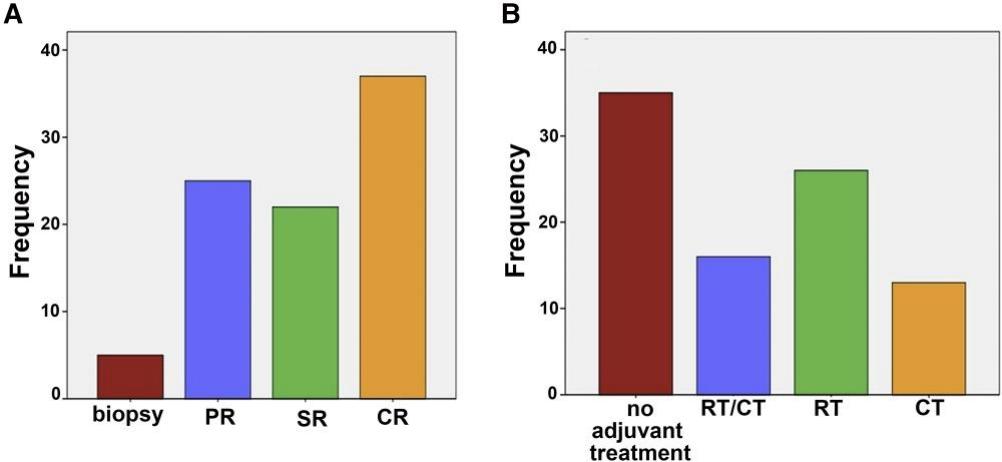
**(A) The extent of surgical resection in the reclassified cohort.** A partial resection of the primary tumor was performed in 25 cases (27.5%), subtotal resection in 22 cases (24.7%), total resection in 36 cases (39.1%), and only a biopsy was performed in five cases (5.5%). (**B**) T**r**eatment **received after surgery in the reclassified cohort.** After surgery, 35 patients (38.5%) did not receive any adjuvant treatment, 16 patients (17.6%) were treated with both radiotherapy and chemotherapy, 26 patients (17.6%) were treated with radiotherapy alone and 14 patients (15.3%) were treated with chemotherapy alone. PR, partial resection; SR, subtotal resection; CR, complete resection; CT, chemotherapy; RT, radiotherapy.

A partial resection was performed in 25 cases (27.5%), subtotal resection in 22 cases (24.7%), total resection in 36 cases (39.1%), and only a biopsy in five cases (5.5%) (Fig. 2A). No data were available in the clinical report in three cases. After surgery, 42 patients (46.2%) were treated with RT, 29 cases (31.9%) with CT, and 16 patients with RT and CT (17.6%). A total of 38.5% did not receive any adjuvant treatment (Fig. 2B).

### Survival data in the reclassified cohort

**Figure 3:**
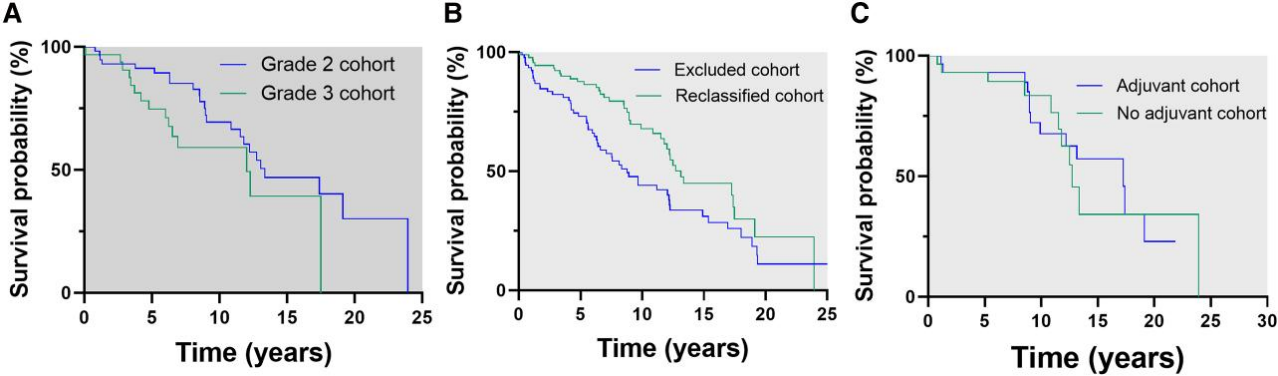
**(A) Median overall survival for Grade 2 and Grade 3 oligodendrogliomas in the reclassified cohort.** There were no significant differences in the median OS between Grade 2 and Grade 3 oligodendrogliomas in the reclassified cohort; 13.35 years (95% CI 8.27–18.44) for Grade 2 oligodendrogliomas (59 cases, 64.8%) and 12.01 years (95% CI 5.66–18.35 years) for Grade 3 (32 cases, 35.2%) oligodendrogliomas (*P* = 0.089). (**B**) Overall survival analysis of patients in the reclassified cohort with the cohort that was excluded from reclassification that did not comply with the molecular criteria. The median OS of the reclassified cohort (91 patients) was significantly longer compared to the series of cases did not meet the reclassification criteria (IDH mutated and 1p/19q codeleted) termed the ‘excluded cohort’ (91 patients); 13.35 years (95% CI 7.97–18.74) for the reclassified cohort and 7.529 years (95% CI 4.67–10.38) for the excluded cohort (*P* = 0.001). (**C**) Overall survival analysis of patients with Grade 2 oligodendrogliomas, with and without adjuvant treatment from in the reclassified cohort. There was no significant difference in the median OS of Grade 2 patients who received some type of adjuvant treatment (30 patients, 50.85%) compared with those who have not received any type of adjuvant treatment (29 patients, 49.15%); 17.39 years (95% CI 9.11–25.66) and 12.73 years (95% CI 10.81–14.64), respectively (*P* = 0.871). CTX, chemotherapy; RT, radiotherapy; TMZ, temozolomide; PCV, vincristine.

The median OS for the entire reclassified cohort was 12.7 years (95% CI 11.3–14.1) and the median PFS was 6.28 years (95% CI 4.67–7.89). A comparative analysis of OS between cases with Grade 2 and Grade 3 oligodendroglioma did not show any differences in the median OS between Grade 2 and Grade 3 oligodendrogliomas. The median OS for Grade 2 oligodendrogliomas was 13.35 years (95% CI 8.27–18.44) and the median OS for Grade 3 oligodendrogliomas was 12.01 years (95% CI 5.66–18.35 years) *P* = 0.089 (Fig. 3A). A comparative analysis of the difference in median OS between those with oligodendroglioma and those with the initial diagnosis of oligoastrocytoma was not statistically significant; 12.73 years (95% CI 10.8–14.65) for oligodendroglioma versus 13.06 (95% CI 11.43–14.68) for oligoastrocytomas, *P* = 0.699. Frontal location was associated with better survival in the initial cohort and also in the reclassified cohort, 17.39 years in the frontal lobe (95% CI 10.35–24), 13.06 in the temporal lobe (95% CI 10–16), 8.59 in the parietal lobe and 3.77 years (95% CI 2.12–5.42) in other locations *P* = 0.05.

Finally, OS was analyzed in those cases that did not receive any adjuvant treatment (including Grades 2 and 3), which was a total of 35 cases, 29 of them were Grade 2 oligodendrogliomas and 6 were Grade 3 oligodendrogliomas. The median OS for Grade 2 oligodendrogliomas without adjuvant treatment was 12.73 years (95%CI 10.81–14.64) and 12.27 years (95%CI 11.84–12.69) for Grade 3 (*P* = 0.441).

### Multivariate analysis

The multivariate analysis, when all variables are included in the model, a correction is observed, with age becoming statistically significant. In fact, age is the only variable that remains significant in predicting OS, with a hazard ratio of 1.033 and a *P*-value of 0.039.

### Comparison of clinical data from the initial cohort and the reclassified cohort

The clinical data of the initial sample and the sample after reclassification were similar in terms of Grade 2/Grade 3 distribution, sex, age, the extent of resection as well as the type of treatment received after surgery. However, the survival data were different, which is summarized in Table 1.

**Table 1:** Summary of demographic, pathology, treatment, and survival parameters

	Initial cohort: Sample selected by previous 2007 WHO classification based on pathology and morphological parameters	Reclassified cohort: Sample with 2021 WHO based on molecular parameters
**Number of cases**	182	91
**Grade 2/Grade 3 (%)**	65.4/34.6	64.8/35.2
**Gender: Females/males (%)**	38.5/61.5	34.1/65.9
**Age (median)**	44.8 (14–75)	44.67 (22–75)
**Extent of resection: Complete/partial/biopsy (%)**	46.7/41.8/7.1	40.7/52.2/5.5
**Received adjuvant radiotherapy**	50%	46.2%
**Received adjuvant chemotherapy**	32%	31.9%
**Overall survival of the entire cohort (median)**	11.97years	12.7 years
**Oligodendrogliomas Grade 2**	13.06 years	13.3 years
**Median OS**		
**Oligodendrogliomas Grade 3**	6 years	12 years
**Median OS**		
**Progression-free survival of the entire cohort (median)**	5.69 years	6.28
**Oligodendrogliomas Grade 2. PFS**	5.95 years	7.13 years
**Oligodendrogliomas Grade 3. PFS**	4.01 years	4.8 years

Furthermore, a comparison was made between the reclassified cohort and the series of cases that were excluded from the initial cohort because they did not meet the criteria of IDH mutated and 1p/19q codeleted (excluded cohort). The median OS for patients who did not meet criteria (excluded cohort) was 7.529 years (95% CI 4.67–10.38) and for those who met molecular criteria (recalcified cohort) 13.35 years (95% CI 7.97–18.74); *P* = 0.001 (Fig. 3B). In addition, further analyses were performed considering each group of tumour grade separately.

### Analysis by tumour grade of the reclassified cohort of Grade 2 oligodendrogliomas

In Grade 2 oligodendrogliomas, the median OS was 13.35 years (95% CI 8.27–18.44) and the median PFS was 7.13 years (95% CI 5.58–8.68 years).

According to the extent of surgery, although there were clear differences, there was no statistical impact on OS depending on the extent of surgery (complete versus non-complete). The median OS was 23.93 years for cases with complete resection,13.06 years for non-complete resection (95%CI 9.65–16.46); *P* = 0.433 and in cases where only a biopsy was performed the median survival was 6.32 years (6.3–6.33).

For Grade 2 patients who received some type of adjuvant treatment, the median OS was 17.39 years (95% CI 9.11–25.66), and for those who have not received any type of adjuvant treatment 12.73 years (95% CI 10.81–14.64); *P* = 0.871 (Fig. 3C). According to the type of adjuvant treatment for Grade 2 tumours, for patients who received RT alone, the median OS was 17.39 years (95% CI 9.92–24.95), 8.89 years for CT alone, the median OS was not reached for those who received radio-chemotherapy, and 12.73 years (95% CI 10.81–14.64) for patients who received no adjuvant treatment, (*P* = 0.807).

In the reclassified series, of the total 59 cases with Grade 2 oligodendroglioma, 12 (20%) cases met low-risk criteria (<40 years with complete resection) and 47 cases had high-risk criteria (79%). For patients considered high-risk Grade 2 oligodendroglioma who had not received adjuvant treatment, the median PFS was 8.44 years and the median OS was 12.73 years (95% CI 9.4–16.05). For low-risk Grade 2 oligodendrogliomas (<40 years with complete resection), the median PFS was 6.19 years and the median OS was not reached.

### Overall results of clinical data of Grade 3 oligodendrogliomas in the reclassified cohort

In Grade 3 oligodendroglioma, the median OS was 12.01 (95% CI 5.66–11.35) years and the median PFS was 4.8 years (95% CI 2.55–7.06). There was no impact on OS depending on the extent of surgery (complete versus non-complete). The median OS was 12.27 years (95% CI 5.64–18.89) for those patients with non-complete resection and was not reached for complete resection (*P* = 0.621). In terms of the type of postoperative treatment, the median OS of patients who did not receive adjuvant treatment was OS 12.27 years (95% CI 11.84–12.69) and was not reached in those with adjuvant treatment. By type of postoperative treatment, the median OS was 6 years for RT alone (95% CI 2.82–9.18) and was not reached in patients who received CT alone or RT and CT. Indeed, median OS was inferior in patients treated with RT alone (monotherapy) compared with other alternatives, 6 versus 12.27 years (95% CI 7.44–13.20), (*P* = 0.004) and on the other hand, a better OS was found in patients who received CT as part of their treatment (chemo alone or chemoradiotherapy) compared with other options, 6.51 months versus not reached (*P* = 0.011).

### Treatment at first progression

**Figure 4:**
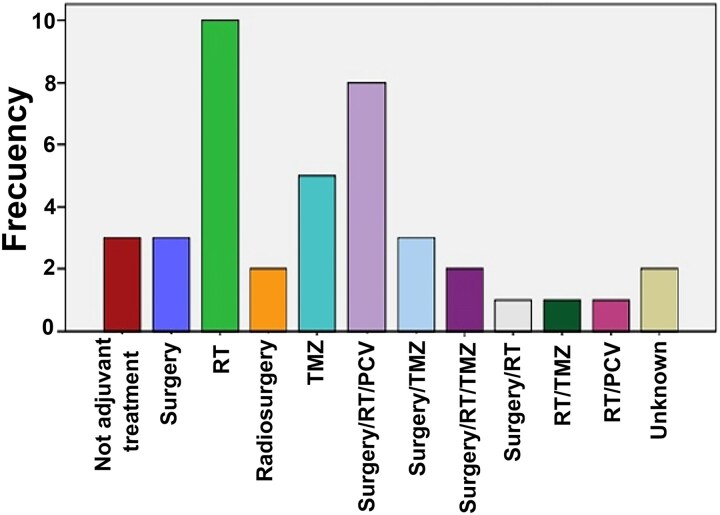
**Treatment received by patients at the time of the first disease progression.** At first progression in the reclassified cohort, three patients received no adjuvant treatment; three patients received surgery alone; 10 patients received radiotherapy alone; two patients received radiosurgery; five patients received chemotherapy alone (TMZ); eight patients received combined surgery, radiotherapy, and PCV; three patients received combined surgery and radiotherapy; two patients received combined surgery, radiotherapy, and TMZ; one patient received combined surgery and radiotherapy; one patient received combined radiotherapy and TMZ; one patient received combined radiotherapy and PCV; and data are not available for two patients. CTX, chemotherapy; RT, radiotherapy; TMZ, temozolomide; PCV, vincristine.

The treatment received by patients at the time of first progression was analyzed in the reclassified cohort: 20% underwent a surgical procedure (some cases also received RT and/or CT); 23% received RT plus another type of treatment (surgery and/or CT); 7.7% only surgery; 12.1% only radiotherapy; 7.7% only chemotherapy (temozolomide); 8.8% received no further treatment (Fig. 4). The median survival after the first progression for the total cohort after reclassification was 6.041 years (95% CI 3.49–8.59). For Grade 2, this was 7.16 years (95% CI 4.79–9.54), and 3.62 years (95% CI 1.66–5.59) for Grade 3 tumours. On the other hand, the evolution in subsequent progressions was analyzed: the median time to progression from the first to the second progression was 3 years and the median time to progression from second to third progression was 1.25 years.

In addition, the possible transformation to high-grade of the gliomas initially diagnosed as Grade 2 was evaluated in this study. Progression to a higher level of malignancy was considered to be present if there was a new contrast uptake and/or if this change in grade was histologically confirmed after relapse surgery. The percentage of malignancy to those considered to have progressed was 54% (21 cases with malignancy data out of 39 with progression data). In those that become malignant, the median time to malignization was 5.11 years.

### Reclassification of tumours considered as oligoastrocytomas by the pre-2021 classification

Of the initial cohort, a total of 40 cases were considered oligoastrocytoma according to the histological diagnosis before 2021 WHO classification and 12 cases (13.2%) of the 91 cases that met the 2021 WHO criteria for oligodendroglioma. Therefore, of the total number of patients with oligoastrocytoma by the previous WHO classification, 30% of the cases (12 of 40) were defined as oligodendroglioma by the 2021 WHO molecular criteria.

## Discussion

### Clinical data of the initial cohort

Oligodendrogliomas are rare CNS tumours^1^; five new cases were diagnosed per year in our series during 37 years, from 1977 to 2014.^4,5^ In our series, these tumours were more frequent in males (60% of cases), the predominant localization was in the frontal lobe (60% of the cases), followed by the temporal and parietal lobes, and the median age at diagnosis was 44.8 years. The entity previously known as oligoastrocytomas accounted for 22% of the total cases. The descriptive data of the series coincides with other reported studies.^4,6,7^

Regarding treatment, the extent of resection reported by Schieie *et al.*, showed similar data to our study, reporting a complete resection in 44% of cases and a biopsy in 13%. Adjuvant chemotherapy was administered in 45% of patients, and radiotherapy in 26%.^4^

In terms of survival, data from the initial cohort of our study (median OS of 13.06 years in Grade 2 oligodendroglioma and 6 years in Grade 3) are also consistent with previous publications, where a median survival of more than 10 years was reported for Grade 2 and 6.3 years for Grade 3 oligodendroglioma^4,8-10^ in a retrospective study by Lassman *et al*.^11^

The data are also consistent with the results of randomized clinical trials, where the median OS in patients without molecular selection was 13 years in Grade 2 oligodendrogliomas in the RTOG 9802 study (RT followed by PCV arm).^12^ For Grade 3 oligodendrogliomas, the median OS was 3.52 years in the EORTC (RT arm followed by PCV)^13^ and 4.7 years in the RTOG 9402 study.^14^

Finally, the analysis of our series showed a statistically significant difference in OS between patients with Grade 2 and Grade 3 oligodendroglioma in the initial cohort classified by morphological criteria, which is also consistent with previously published studies.^10^

### Clinical characteristics of the global entire reclassified cohort (Grade 2 and Grade 3)

The clinical data and recommended treatment were similar in the initial and reclassified cohorts. However, in terms of survival data, we found some differences.

The median OS for the entire series was 12.73 years, 13.3 years for Grade 2 oligodendrogliomas, and 12 years for Grade 3 oligodendrogliomas. This is an interesting and striking aspect that we want to highlight since, when comparing these data, OS between Grade 2 and Grade 3 oligodendrogliomas in the reclassified cohort, the differences were not statistically significant. This point marks a difference from what we had observed in the initial cohort, where we had seen a statistically significant difference in OS between Grade 2 and Grade 3 tumours.

Thus, molecular grading modifies the impact of grade on survival. Furthermore, recent studies also indicate the absence of differences between Grades 2 and 3 when the molecular study of IDH mutation and codeletion of 1p/19q are applied to the diagnosis.^15,16^ Moreover, statistically significant differences were found between the reclassified cohort and the excluded cohort (median OS in the reclassified cohort was 13.35 and in the excluded cohort 7.52 years) indicating the importance of molecular characterization to better characterize the true oligodendroglial tumours and define the prognosis of these patients.

I**n Grade 2 oligodendroglioma**, regarding the extent of surgery, other studies have reported a higher percentage of biopsies and a lower percentage of complete resection than that reported here,^12,17^ which may be related to the retrospective nature of our series. In our series, the extent of surgery was associated with longer survival (PFS and OS) for patients with complete resection, but without reaching statistical significance. Likewise, in a systematic review of the extent of surgery from 2015, the authors reported an impact on PFS and provided a level of evidence II to recommend surgery based on total or subtotal resection instead of a biopsy, whenever possible. Furthermore, they found an impact of the extent of surgery on OS, with Level III evidence.^18^

In terms of survival of Grade 2 oligodendrogliomas in the reclassified cohort, our results are similar to the phase III trial RTOG 9802, which showed a median OS of 13.3 years for the arm of RT-CT. In fact, the data from our cohort were superior to the control arm (with RT alone), which could be related to a better-characterized oligodendroglioma series after using molecular criteria, even though the patients of our cohort had a less intensive treatment than those in the clinical trial (in our cohort only 10% had CT-RT). In this Grade 2 cohort, the majority (79%) were high-risk cases, as defined in RTOG 9802 (>40 years or incomplete resection). We analyzed data from those cases without adjuvant CT: the median PFS was 4.9 years for low-risk cases and 6.19 years (95% CI 4.18–8.21) versus 8.44 years (95% CI 1.92–14.96) for high-risk (median OS was not reached). These data indicate that clinical criteria may not be sufficient or lose their prognostic impact for patients with a diagnosis based on current molecular criteria.

In **Grade 3 oligodendroglioma**, there was a lower percentage of biopsy procedures in our cohort compared to other studies.^11,13,19,20^ The postoperative treatment data in our study highlight the variability in the treatment of this disease over the last decades, which has also been reflected in other studies, such as the results of a survey of members of the Neuro-Oncology Society in the United States by Abrey *et al*. in 2006.^21^ Furthermore, it should be noted that it was not until 2013 that the final results of the EORTC 26951 and RTOG 9402 phase III studies were known, which indicated a benefit in OS for codeleted patients when treated with RT and chemotherapy with a PCV schedule versus radiotherapy alone.

Regarding survival data, in the EORTC 26951,^13^ the median OS was 3.52 years for patients treated with chemoradiotherapy versus 2.55 years for those treated with radiotherapy (*P* = 0.018). However, when the subgroup of codeleted cases was analyzed, OS was 9.3 years for those treated with RT and not reached for those treated with chemoradiotherapy (*P* = 0.059). In the RTOG 9402 study,^22^ the median OS was 4.6 and 4.7 years, however, when the cases that had 1p/19q codeletion were analyzed, the median OS was 14.7 years for patients treated with chemoradiotherapy and 7.3 years for those treated with radiotherapy alone. In NOA04^20^ in the codelected patients, OS was not yet achieved for PCV or the RT arm. Finally, the large retrospective series of Grade 3 oligodendroglioma cases by Lassman *et al.* reported a median OS was 8.5 years and PFS was 4.5 years in codeleted patients.^11^ Therefore, the results found in our series are similar to those of the more intensive treatment arm with RT and PCV in the RTOG 9402 and EORTC 26951 studies, and better than the monotherapy arm, although only 31% received CT-RT and 18.8% did not receive adjuvant treatment in our series. These data underline, once again, the prognostic importance of a good characterization of these tumours.

Regarding Grade 3 oligodendrogliomas, a trend towards a better evolution with the administration of postoperative CT (with or without RT) was also observed in our cohort. This was also observed in the study by Lassman *et al.*,^11^ thus, underlining the importance of adjuvant CT in Grade 3 oligodendrogliomas.

### Multivariate analysis

In our complete reclassified series of oligodendroglioma, age is an independent prognostic factor for OS. We demonstrated this in a multivariate analysis that also considered other variables such as tumour grade, extent of resection, and adjuvant treatment. This result is consistent with the available data from a large a multicenter study from the French POLA network in which only age was independently correlated to survival.^23^

### Characteristics, treatment, and evolution of the first progression

The choice of treatments at disease progression is often linked to the treatments received at diagnosis, therefore we observed different treatment approaches in our cohort. Similar to our results, the RTOG 9802 study also reported surgical intervention rates of around 20% at the time of first progression,^12^ furthermore, other published series have reported different treatment approaches, without a standardized choice.^7^ The median survival for the entire cohort after the first progression was 6 years (for Grade 2 tumours 7.1 and, for Grade 3 tumours 3.62 years). Our data also indicates that the median survival shortens with each new progression, such that the median survival was 3 years from the first to the second progression and 1.25 years from the second to the third progression, which is probably related to a greater aggressiveness of the tumour and/or a lesser response to the treatments administered. Progression to a high-grade tumour (from initial low-grade tumours) was reported in 54% of the tumours that progressed at some point in the follow-up, which is consistent with data reported in the RTOG 9802 study^12^ and 33% in some other studies.^7^ The median time until this grade increase occurred was 5.11 years, which is in line with other studies.^24^

### Reclassification of tumours considered as oligoastrocytomas in the pre-2021 WHO classification

Sahm *et al.* performed a reclassification of a series of oligoastrocytoma tumours and found that 1p/19q codeletion and mutation of ARTX and p53 were mutually exclusive, confirming that these tumours with mixed histologic features were ultimately characterized at the molecular level as either oligodendroglioma or astrocytoma.^25^ They reported that 72% of the cases initially considered oligoastrocytoma had molecular features typical of oligodendroglioma and 25% of astrocytoma, which differs from our study (30% of all oligoastrocytic tumours were characterized as oligodendrogliomas). However, other studies have reported results closer to ours, such as Kim *et al.* (with 13.7% of cases reclassified to anaplastic oligodendroglioma).^26^

The median survival for tumours considered oligodendrogliomas was 12.73 years and 13.06 for those reclassified from initially considered oligoastrocytomas (*P* = 0.699). Therefore, these data reinforce similar biological behaviour once reclassified. In any case, the small number of patients analyzed must be taken into account. Furthermore, it should be taken into account that this is a retrospective analysis, with the biases intrinsic to this type of study. On the other hand, the size of the series, although it is a high number for such a rare disease as oligodendroglioma, is still a small size for statistical analysis.

## Conclusions

The molecular classification of oligodendroglial tumours implies a more accurate selection of oligodendroglioma, with a better prognosis, regardless of the grade and treatment received.

## Supplementary Material

fcaf265_Supplementary_Data

## Data Availability

Additional data and materials may be made available from the corresponding author upon request.
