# Supplementary material for: Oligodendrogliomas: findings after classifying the same cohort using pre- and post-World Health Organization (WHO) 2021 criteria
Source: Brain Commun. 2025 Aug 1;7(4):fcaf265. doi: 10.1093/braincomms/fcaf265 (PMC12314594; doi:10.1093/braincomms/fcaf265)

**Supplementary Figure 1.** Univariate overall survival analysis based on age (a), grade (b), degree of resection (c), and adjuvant treatment (d) was performed using the log-rank test and illustrated with Kaplan–Meier plots.


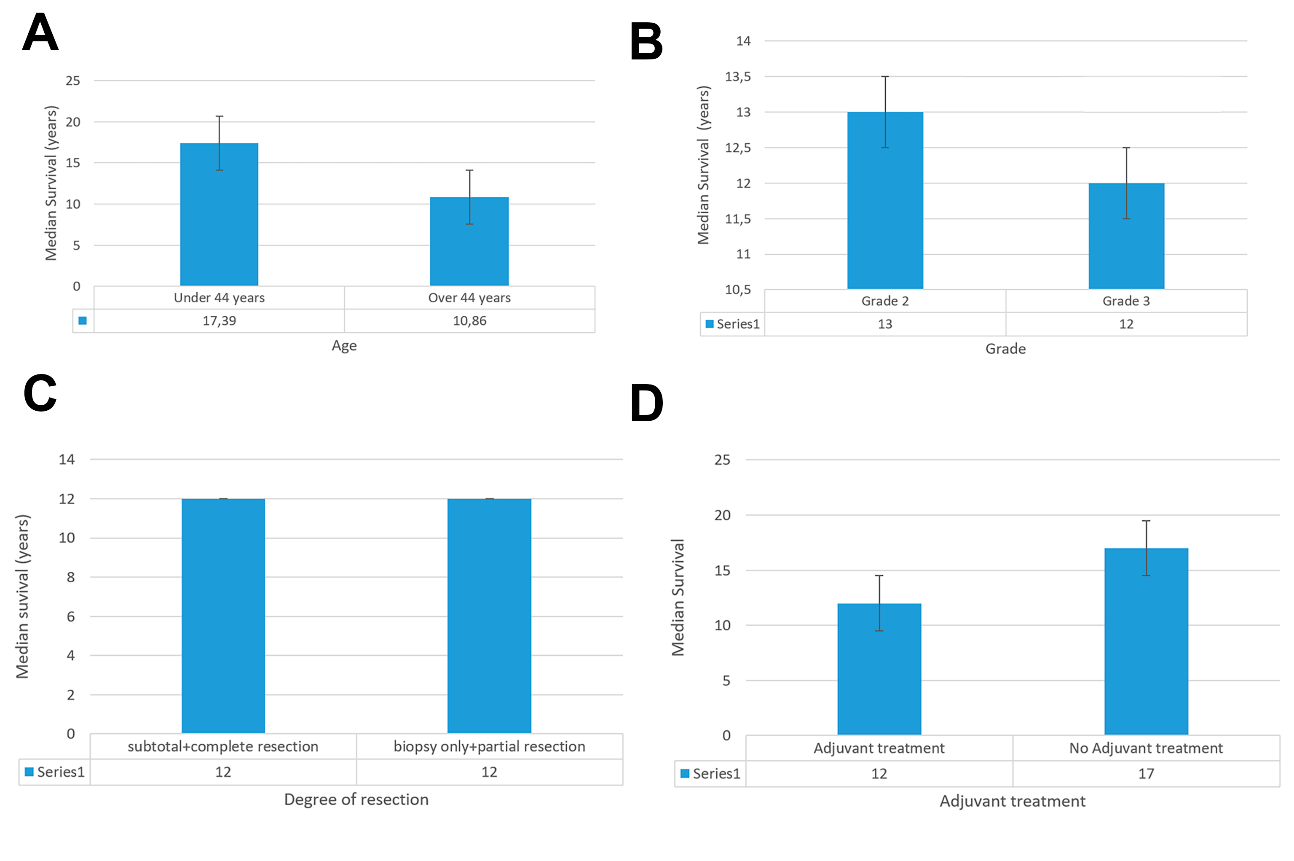

Supplement: fcaf265_Supplementary_Data [file fcaf265_supplementary_data.docx]
